# Magnetic Resonance Imaging in Pregnancy with Intrauterine Growth Restriction: A Pilot Study

**DOI:** 10.1155/2019/4373490

**Published:** 2019-11-16

**Authors:** Serafina Perrone, Antonino Santacroce, Giuseppe de Bernardo, Maria Gabriella Alagna, Salvatore Francesco Carbone, Irene Paternò, Giuseppe Buonocore

**Affiliations:** ^1^Department of Molecular and Developmental Medicine, University of Siena, Italy; ^2^Department of Mother's and Child's Health, Poliambulanza Foundation Hospital Institute, Brescia, Italy; ^3^Department of Radiological Sciences, University of Siena, Italy

## Abstract

**Objective:**

Intrauterine growth restriction (IUGR) is a major cause of late stillbirth, though not all compromised babies remain small or are considered growth restricted as pregnancy progresses. Fetal Magnetic Resonance Imaging (f-MRI) represents a second-line tool to study pregnancies with IUGR fetuses. The aim of our study was to evaluate the usefulness of f-MRI on predicting fetal growth and the offspring's perinatal respiratory outcome.

**Design:**

All f-MRI performed between 2014 and 2016 in Siena were analysed. Pregnancies with IUGR (Study group (SG)) were recruited together with a control population (Control group (CG)), coupled for gestational age (GA) at the time of f-MRI (mean GA 31 wks). Neonatal information was collected. The f-MRI protocol consisted of T2w images. Six regions of interest (ROI) were placed as follows: 2 on the lung, 2 on the liver, and 2 on the amniotic fluid. The signal intensities (SI) of each ROI were measured. The SI lung to liver ratio (SI lung/liver) and SI lung to amniotic fluid ratio (SI lung/amniotic fluid) were obtained for each fetus. Each ratio was compared between SG and CG. Therefore, SG was divided into two subgroups: adequate and small for gestational age (AGA and SGA) newborns. All measurements were related to offspring's perinatal respiratory outcome.

**Results:**

SI lung/liver was linearly related with GA at the time of f-MRI and with EFW. SI lung/amniotic fluid was significantly higher in SG than in CG (*p* = 0,014). In contrast, among SG, lower values of SI lung/amniotic fluid were found in the SGA compared to AGA (*p* = 0,036). The days of oxygen supply were higher in the SGA subgroup than in the AGA subgroup (*p* = 0,028).

**Conclusions:**

SI lung/liver increases with fetal lung maturation and appears to be useful to estimate intrauterine fetal growth. SI lung/amniotic fluid seems to be a reliable predictive index to distinguish the IUGR fetuses that can recover their growth from those that were born SGA. f-MRI represents a promising frontier to predict IUGR fetus outcome, thus contributing to ameliorate the perinatal management.

## 1. Introduction

Complex and dynamic interactions between maternal, placental, and fetal environments are involved in ensuring normal fetal growth. The impairment of this system may lead to intrauterine growth restriction (IUGR) [1]. If in utero alterations persist, fetuses can be born small for gestational age (SGA). Thus, the terms IUGR and SGA, although related, are not synonymous: IUGR defines deviation from the expected (potential) fetal growth pattern; SGA describes an infant whose weight at birth is lower than a predetermined cut-off weight. The cause may be pathologic, in the case of an infant with IUGR, or non-pathologic, in the case of an infant who is small but healthy [2].

Being born SGA is associated with many short-term complications like increased mortality, lung disease, hypotension, necrotizing enterocolitis, poor thermoregulation, hypoglycaemia, and polycythaemia. In addition, long-term complications, like insulin resistance, type II diabetes mellitus, cardiovascular disease, neurodevelopmental and stature delay, and cognitive and behavioural problems, may occur in SGA newborns later in life [3].

There is no consensus on which fetal monitoring modalities should trigger delivery in fetal growth restriction between 32 and 36^+6^ weeks, and practice varies widely worldwide [4, 5]. The consequences of inappropriately early or late delivery for perinatal and infant health and maternal morbidity are potentially enormous. Current interest lies in the use of fetal ultrasound (f-US) as the gold standard for screening fetal growth, because it provides real-time images with excellent resolution, repeatable with good cost-effectiveness [6, 7]. However, few useful US parameters are available to predict neonatal outcome and aid in making therapeutic decisions. To discover a fetus at risk of severe IUGR is of paramount importance in the timing of delivery to optimise perinatal outcome.

In the past, Fetal Magnetic Resonance Imaging (f-MRI) was used in pregnancies but fetal anatomy was not adequately visualised with conventional sequences due to long acquisition times [8]. This problem was overcome with the introduction of fast and ultrafast sequences that reduced fetal motion artefacts, in particular T2-weighted sequences. [9] Nowadays, f-MRI is used as a second-line method to study obstetric disorders, fetal anomalies, gravid uterus, and placenta diseases [10, 11]. Moreover, f-MRI is capable of informing on the development of individual organs and their additional maturational changes [12]. For example, f-MRI assessment of the fetal lung leads to specific evaluation of the following: (i) the fetal lung volumetry, used to identify restricted and insufficient lung growth; (ii) the signal intensities (SI), used to analyse the maturational status of the fetal lung; and (iii) the high resolution and tissue contrast, used to offer more detailed information of fetal pulmonary pathologies. These techniques are used in conditions such as oligohydramnios, congenital diaphragmatic hernia, and pulmonary hypoplasia, where studying fetal lung growth and its maturation plays a key role in postnatal survival [13]. In the case of IUGR, placental function and fetal health are usually assessed by ultrasound biometry, Doppler velocimetry measurements, and cardiotocography [4]. f-MRI is used infrequently for the diagnosis of IUGR outside research settings.

The aim of this work was to evaluate the usefulness of f-MRI in the outcome of IUGR fetus and to study the role of lung intensity signal in predicting perinatal respiratory outcome.

## 2. Materials and Methods

### 2.1. Study Design

All f-MRI performed at “S. Maria alle Scotte” Hospital, in Siena, between May 2014 and October 2016, were analysed. All IUGR pregnancies with gestational age (GA) at the time f-MRI between 29 and 32 weeks were selected (Study group (SG)). Congenital malformations, chromosomal abnormalities, inborn errors of metabolism, outborn birth, and multiple pregnancies were considered the exclusion criteria. The Control group (CG) included fetuses without IUGR, matched for gestational age at the time of f-MRI. The study protocol was approved by the local ethics board. Written informed consent was obtained from the mother before the enrollment.

### 2.2. Population

The estimated fetal weight (EFW) was calculated with Hadlock's method using measurements of biparietal diameter (BPD), head circumference (HC), abdominal circumference (AC), and femur length (FL) of the f-US performed nearest to the f-MRI. IUGR was defined as fetus with AC or EFW below the 10^th^ percentile. A total of 8 pregnancies with IUGR were enrolled in the study, together with a control population of 10. All pregnancies were followed until parturition. At birth, newborns weighing below the 10^th^ centile expected for their GA were considered SGA [14].

In order to discriminate the fetuses that recover their growth from those who were born with a birth weight lower than a predetermined cut-off, the IUGR population was also divided in the adequate for gestational age (AGA) and SGA subgroups.

For each newborn, the following perinatal data were collected: GA and weight at birth; percentiles of weight, length, and head circumference; 1 and 5-minute APGAR score; length of oxygen therapy in days; and maximum FiO₂ levels delivered in the first week of life.

### 2.3. MRI Scans

All f-MRI were performed using a 1.5 T scanner (Signa Excite TwinSpeed HDx, GE Healthcare, Milwaukee, USA) with an eight-channel phased-array surface coil. To prevent supine hypotension syndrome, the examinations were conducted with mothers in a left lateral tilt position.

The imaging protocol consisted of a T2-weighted single-shot turbo spin echo sequences (TR 1000 msec; TE 80 msec; matrix: 224x224; acceleration factor ASSET 2; slice thickness: 3 mm).

**Figure 1:**
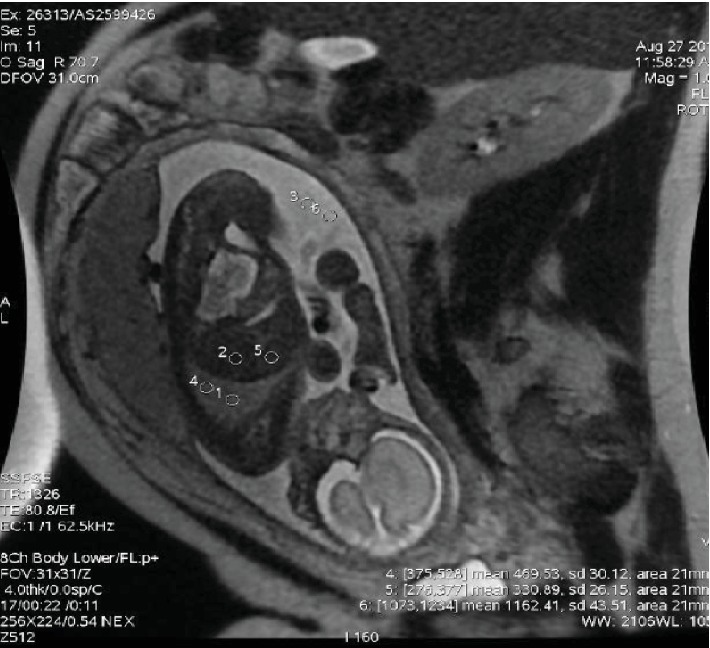
Coronal SSFSE T2-weighted image where 6 ROI (regions of interest were placed: 2 on the lung, 2 on the liver, and 2 on the amniotic fluid).

The image analysis was performed on a workstation (Advantage Windows 4.4, GE Healthcare, USA), and 6 ROIs (Region of Interest) of 200 mm^2^ were placed as follows: 2 ROIs on the lung, 2 on the liver, and 2 on the amniotic fluid (Figure 1). The signal intensities (SI) of lung, liver, and amniotic fluid were quantified, and the average, for each structure, was calculated. As SI of any tissue would depend on the distance from the coil, absolute values are not appropriate to use and therefore a ratio comparing the SI of the lungs with a reference structure at a comparable depth was introduced [15]. The liver and the amniotic fluid were the reference structures used so far, due to the assumption that they are adjacent to the lungs and, more importantly, that they would not change with increasing GA [16, 17]. Therefore, the SI lung to liver ratio (SI lung/liver) and SI lung to amniotic fluid ratio (SI lung/amniotic fluid) were obtained for each fetus.

### 2.4. Statistical Analysis

A descriptive analysis of the population was conducted. For the inferential analysis, SI lung/liver and SI lung/amniotic fluid were correlated to fetus anthropometric measures using Spearman's test. Differences between groups (IUGR vs. Control and AGA vs. SGA) were studied using the Mann-Whitney test.

All the results were also correlated to the subsequent perinatal respiratory outcome in terms of length of oxygen supply in days and the maximum inspired oxygen fraction administered in the first week of life using the Mann-Whitney test. The data were analysed using SPSS v.20.0 software for Windows.

All statistical tests were two-tailed with significance level *p* < 0, 05.

## 3. Results

The population characteristics are shown in Table 1. Regarding the GA at the time of f-MRI or GA at birth, no statistical differences were found between SG and CG. The general mean GA at the time of f-MRI was 31, 6 ± 0, 88 weeks of gestation.

SG had lower EFW and birth weight (BW) than the CG (*p* < 0, 05).

**Figure 2:**
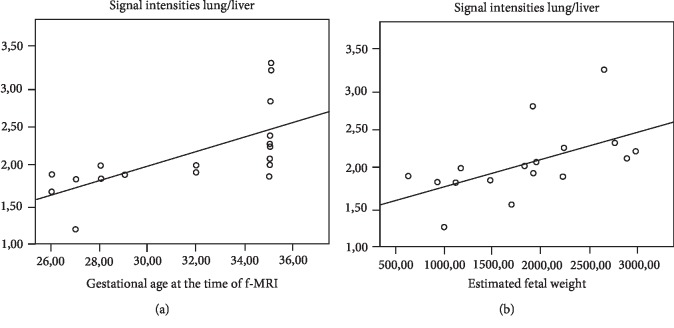
Correlation between SI of the lung/liver with GA (a) and EFW (b) at the time of f-MRI.

SI lung/liver was linearly related to GA at the time of f-MRI (Rho = 0,858; *p* < 0,001) (Figure 2(a)) and with EFW (Rho = 0,794; *p* < 0,001) (Figure 2(b)).

**Figure 3:**
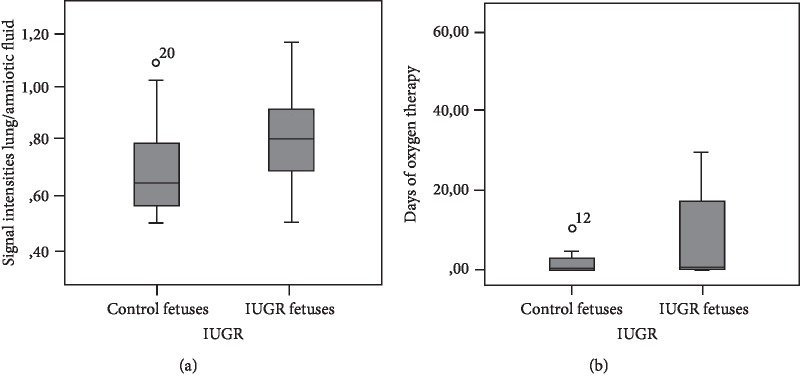
SI lung/amniotic fluid in the IUGR and Control groups (a); days of oxygen therapy in IUGR group (b).

SI lung/amniotic fluid showed no correlation with anthropometric measures but was significantly higher in the SG than in the CG (respectively, 0, 80 ± 0, 06 and 0, 71 ± 0, 06; *p* = 0,014) (Figure 3(a)). SI lung/liver was not significantly different between the groups.

There was a longer duration of oxygen use within the IUGR population (IUGR: 12, 25 ± 8, 13 vs. Control: 1, 9 ± 1, 01; *p* = 0,005) (Figure 3(b)).

**Figure 4:**
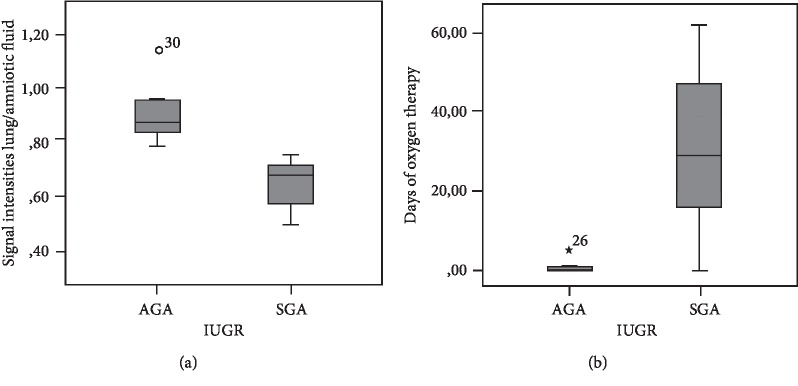
SI lung/amniotic fluid in the SGA and AGA newborns (a); days of oxygen therapy in SGA and AGA newborns (b).

In contrast, among IUGR fetuses, lower values of SI lung/amniotic fluid were found in the SGA population in comparison with AGA (respectively, 0, 63 ± 0, 07 and 0, 9 ± 0, 06; *p* = 0,036) (Figure 4(a)). SGA newborns received oxygen therapy longer than AGA (30, 66 ± 18, 52 vs. 1, 2 ± 0, 96 days; *p* = 0,028) (Figure 4(b)).

## 4. Discussion

The fetus with IUGR represents a public health problem, and it is the second most common cause of perinatal mortality and morbidity, after the preterm delivery. It is important to enhance the fetal management techniques in order to ameliorate IUGR prenatal and postnatal outcome.

This is the first study that investigates the usefulness of the f-MRI in the evaluation of IUGR perinatal outcome, demonstrating that f-MRI parameters may be used to estimate fetal growth trends. Significant associations between the SI lung/liver and gestational age and EFW at the time of f-MRI were found in our study [16].

Given that the reference structure does not change during pregnancy, the increase of the SI value in the ratio lung/liver means that the lung increases its water content during gestation. In fact, fetal lung fluid secretion increases during gestation and correlates with progressive pulmonary development and maturation: when the volume of fetal lung fluid is abnormally small, lung hypoplasia occurs, and in contrast, excess fetal lung fluid results in lung hyperplasia [17, 18]. The SI amniotic fluid/lung allowed to distinguish the IUGR fetuses that would recover their growth from those that would have been born SGA. The reduction in the SI ratio in SGA infants could be due to a premature resorption of pulmonary fluids due to fetal stress or an adverse intrauterine environment that did not determine the correct achievement of growth potential. It would be also plausible that IUGR fetuses that do not regain growth and are born SGA have a primitive lung development disorder.

Although secretion of fluid into the lung lumen is vital for growth of the fetal lung, this fluid must be removed at birth to help the newborn convert from “liquid breathing” to “air breathing” mode. Fetal lung secretion declines in the last few days before the labour [19], and the lung epithelium absorption begins. This process is mediated through the activation of airway epithelial sodium channels (ENaC). In humans, postnatal ENaC expression is gestational age-dependent, and its activity correlates with lung compliance. It is therefore likely that in the human newborn infant ENaC is also important for physiologic postnatal adaptation. Low pulmonary expression or activity of ENaC in the perinatal period may cause delayed clearance of lung fluid and thereby contribute to the development of respiratory distress in both term and preterm infants. [20] In our study, SI lung/amniotic fluid was significantly higher in the IUGR group than in the Control group. This increase in water content can explain the different respiratory outcomes of the two groups; in fact, the length of oxygen therapy was significantly higher in the IUGR group.

Unexpectedly, SI lung/amniotic fluid was found to be significantly lower in the SGA than in AGA subgroups and the days of oxygen therapy were higher in the SGA newborn than in the AGA subgroup. According to our knowledge, this result has never been reported in the literature.

Epinephrine, O₂, glucocorticoid, and thyroid hormones interact to stimulate Na^+^ absorption by increasing Na^+^ pump activity [24]. IUGR fetuses are overexposed to the effects of glucocorticoids [26]: their increase during fetal development may contribute to facilitate the absorption of the pulmonary fluid.

IUGR fetuses that do not recover their growth are exposed to greater in utero stress condition, and the acceleration of pulmonary development may result to a worse respiratory outcome due to a premature activation of the absorption phase that impairs the normal lung development.

## 5. Conclusions and Future Perspectives

The data analysis allowed the identification of a set of f-MRI parameters useful to predict perinatal outcome of IUGR fetuses.

SI lung/liver was related to anthropometric measurements and could become a potential index of fetal lung maturation, thus useful to estimate intrauterine fetal growth. In contrast, SI lung/amniotic fluid could be an index capable to predict the respiratory outcome and to distinguish the IUGR fetuses that can recover their growth, from those that are born SGA.

f-MRI represents a promising frontier to predict IUGR fetal outcome, thus contributing indications about how to treat fetal growth restriction in utero. Accordingly, fMRI might still be systematically performed when IUGR is diagnosed, in order to provide information relevant for growth and respiratory outcome of the fetuses, needing a closer follow-up.

fMRI could be a reliable aid for managing neonatal intensive care treatment and parent counselling since a normal/subnormal MRI would offer a reassuring prognosis.

Further studies with a larger number of fetuses and neonates are needed, particularly involving the addition of clinical, biological, electrophysiological intensive care data. Research efforts in this field will provide some definitive answers to establish the optimal timing of delivery of IUGR, in order to achieve their best outcome.

## Figures and Tables

**Table 1:** Clinical characteristic of population study.

	Total *n* = 18	SG *n* = 8	CG *n* = 10
Maternal age	33, 35 ± 4, 1	35, 5 ± 3, 5	32, 2 ± 4, 2
GA at f-MRI (weeks)	31, 47 ± 3, 79	30, 57 ± 4, 19	32, 1 ± 3, 57
EFW (g)	1827, 17 ± 720,45	1533, 14 ± 780,16	2033 ± 634,56
GA at birth (weeks)	37, 23 ± 2, 81	36, 42 ± 3, 64	37, 8 ± 2, 09
Weight at birth (g)	2800, 0 ± 635,64	2430, 71 ± 800,48	3058, 5 ± 332,87^∗^
Weight percentile	39, 47 ± 26, 41	19, 07 ± 12, 13	53, 75 ± 24, 33
Length at birth (cm)	47, 11 ± 3, 75	45, 0 ± 4, 47	48, 60 ± 2, 41
Length percentile	36, 88 ± 25, 52	18, 50 ± 12, 2	49, 75 ± 24, 73
Head circumferences (cm)	33, 04 ± 2, 56	31, 58 ± 3, 45	34, 07 ± 0, 96^∗^
Head percentile	44, 26 ± 27, 06	28, 29 ± 22, 44	55, 45 ± 25, 06
APGAR 1	8, 7 ± 1, 26	8, 71 ± 1, 25	8, 7 ± 1, 33
APGAR 5	9, 23 ± 1, 2	9, 28 ± 1, 49	9, 2 ± 1, 03
Max FiO_2_ during the first week of life	0, 24 ± 0, 06	0, 25 ± 0, 07	0, 24 ± 0, 05
Days of oxygen therapy	6, 58 ± 16, 34	13, 28 ± 24, 64	1, 9 ± 3, 2

^∗^ *p* < 0.05.

## Data Availability

Data are available on request.
